# Volunteers' Demographics That Affect the Human-Dog Interaction During Walks in a Shelter

**DOI:** 10.3389/fvets.2021.699332

**Published:** 2021-09-07

**Authors:** Hao-Yu Shih, Mandy B. A. Paterson, Nancy A. Pachana, Clive J. C. Phillips

**Affiliations:** ^1^School of Veterinary Science, University of Queensland, Gatton, QLD, Australia; ^2^Royal Society for the Prevention of Cruelty to Animals Queensland, Brisbane, QLD, Australia; ^3^School of Psychology, University of Queensland, St Lucia, QLD, Australia; ^4^Curtin University Sustainability Policy Institute, Bentley, WA, Australia

**Keywords:** demographics, dog, leash tension, dog-walking, shelter, canine behaviour, human behaviour, human-dog interactions

## Abstract

Different people relate to dogs in different ways. We investigated differences between volunteers in their behavioural interactions with shelter dogs when they were walked on a leash. Cameras were used to record and quantify the behaviour of volunteers and a leash tension metre was used to measure pulling by both volunteers and shelter dogs. Effects of volunteers' age, body height, educational level, marital status, and experiences of living and working with dogs, and living with children, were examined. Older volunteers talked to the dogs more often during the walk than younger ones. Taller volunteers had reduced physical contact with dogs, and dogs pulled more frequently on the leash while walking with them. Volunteers with a postgraduate degree more frequently praised dogs and rewarded dogs with food and used more body language in the form of hand gestures and physical contact. Married and partnered volunteers more often praised dogs, while separated/divorced or widowed volunteers initiated more frequent physical contacts. Dogs pulled less when walking with volunteers who had experience of living with dogs, and these volunteers interacted with dogs using fewer verbal and body languages. Finally, those living with children more frequently communicated with dogs using body language (e.g., hand gestures and physical contact). We conclude that shelters should carefully consider volunteers' demographics when selecting them to walk dogs with various behavioural characteristics.

## Introduction

Dogs (*Canis familiaris*) have become an important part of modern society. It is estimated that 38% of U.S. (1) and 40% of Australian (2) households own dogs. Studies investigating human-dog interactions involve interspecific communicative skills (3), training techniques (4), stress modulation (5) and benefits of shared activities, such as dog walking (6). Canine factors identified to affect the human-dog relationship include morphological traits (7), age (8), breed (9, 10), and behaviour (9). For humans, influencing factors include training techniques used (11), personality (12) and other demographic factors [e.g., gender (13) and socioeconomic status (14)].

Different types of people interact differently with dogs (12, 15, 16). More educated people better identify the signs of stress in dogs (17); use more reward-based training techniques (16) and develop closer relationships with their dogs (18). However, there are some contrary results from other studies: that less veterinary care is given to dogs owned by more highly educated people (19), that less educated owners have a stronger human-dog attachment (20). Older owners have been reported to be less attached to their dogs (21) and provide their dogs with less social support in stressful situations (15). Young adults are more likely to play with their dogs, but they are also more likely to jerk on the leash to correct their dogs (22). Owners who are single give more attentive care to their dogs, and owners without children in their family develop a stronger attachment with their dogs than those with children (19). Hence, family size is positively correlated with behavioural issues in dogs, such as disobedience and aggression, potentially due to a lack of time for the dog's training, inconsistent training, or tolerance of undesirable behaviours (23). Dogs are less likely to develop behavioural issues if their owners have previous dog-ownership experiences and spend more time with their dogs (24). Finally, Wells and Hepper (25) reported that dogs were more stressed while interacting with men, probably because men are on average taller than women, making dogs perceive men to be more intimidating. However, inconclusive results were found that dogs of male owners had lower cortisol reactivity compared to dogs of female owners (26).

Most research exploring the relationship between human demographics and human-dog interactions was investigated in owners and pet dogs. Research about human-dog interaction in animal shelter settings is limited, despite potential for positive interactions, such as petting (27), training (28), and on-leash walking (29), to benefit the shelter dog's welfare (5). Compulsory leash use policies have been implemented around the world when walking dogs outdoors in order to protect wildlife (30), decrease disease transmission (31), and reduce dog biting (32) and traffic injuries (33). However, leash pulling is one of the most common issues reported in shelter dogs within 1 month post-adoption (34). It can be detrimental to the health of the dog (35) and is related to handlers' musculoskeletal injuries (36). Also, it is equally important that how handlers hold the leash because owners jerking on the leash can cause severe health concerns to the dogs (37). Therefore, a good human-dog interaction on the leash involves reduced canine stress related behaviours, decreased pulling on the leash by both dogs and humans and improved human satisfaction.

In this study, we researched human-dog interactions in an animal shelter focusing on the influence of human demographics and the human-dog dyad when volunteers walked dogs on a leash. In addition to using video recording, a canine leash tension metre was used to measure the tension on the leash, using a three-axis accelerometer to differentiate the directional forces (38, 39).

It was hypothesised that, as in the household environment, highly educated volunteers would be more likely to praise and offer food rewards when interacting with shelter dogs (16), but would be less likely to be satisfied with the interaction because they are potentially less anthropomorphic (40). We also hypothesised that volunteers with experience of living with dogs would communicate with dogs more effectively by pulling on the leash less and using fewer verbal and physical cues (24).

## Materials and Methods

### Ethics Statement

This study was approved by the Human Research Ethics and Animal Ethics Committees (Approval numbers: 2018001570 and SVS/400/18, respectively) of the University of Queensland.

### Study Site

The research was conducted at the Royal Society for the Prevention of Cruelty to Animals, Queensland (RSPCA, QLD) shelter. The shelter environment, housing schedule and study site have been fully described previously (41).

### Subjects

#### Dogs

This study involved 111 shelter dogs and 74 volunteers, with each volunteer walking 5 different dogs on a leash, yielding 370 walks. Dogs were classified into four levels by RSPCA staff based on their behaviour on the leash. Level 1 dogs walked on a loose leash most of the time. Level 2 dogs pulled on the leash during the walk occasionally and had more undesirable behaviours. Level 3 dogs tended to pull on the leash fiercely due to excitement or timidity. Level 3+ dogs did not necessarily pull on the leash any harder, but they had severe behavioural issues, such as overt excitement or fearfulness (41). In addition, all participating dogs had undergone an RSPCA behavioural assessment (42).

#### Volunteers

Volunteers had gone through a series of standardised training programs in four stages allowing them initially to walk level 1 dogs. With each learning stage they learnt how to walk the more challenging dogs (level 3 and 3+ dogs). Dogs were assigned to volunteers by RSPCA staff for a daily walk based on the volunteer's training level, and volunteers could only walk dogs that had the same or lower behavioural level than their training level.

### Canine Leash Tension Metre

The custom designed canine leash tension metre (sampling rate: 10 Hz; measuring range: 0–100 kg-force; resolution: 100 g-force) [RobacScience Australia (39)] measured the force exerted on the leash and detected the direction of the pull (handler vs. dog). One end of the device could be held by the handler and the opposite end was connected to a l.4-metre-long commercial dog leash (Rogz Snake Lead), which was attached to both the collar and the harness at the front of the dog's chest [for further details see (39)]. This article focuses on human demographics. Results of other human and dog factors, including volunteers' personality and gender, the sex, size and behavioural assessment results of the dogs, have been reported (39, 41, 43, 44).

### Study Design

Volunteers completed a consent form for the research, a demographic questionnaire (Table A1) and a NEO Five-Factor Inventory personality test (45). Dogs were matched to volunteers of the right training level by RSPCA staff, and each volunteer was instructed to walk 5 different dogs in the designed area using the canine leash tension metre. All walks were video recorded using a GoPro headset (GoPro Hero 7 Silver, GoPro®, San Mateo, CA, USA) mounted on the volunteer's head and an i-Phone 7 (Apple Inc., Cupertino, CA, USA) held by the experimenter following 10 m behind. All walks started from the kennel, progressed along the designated pathway and ended with returning to the kennel. Only the interactions when dogs were on the designated pathway were recorded. The ground of the designated pathway had several sections with different coverings. The first 40% was covered with gravel, followed by 20% of concrete, then 5% on wooden boards and finally the last 35% was covered with earth. Equipment and infrastructure provided for added stimulation and play including two bridges, two dog jumps, some tennis balls and some hanging plastic milk bottles.

At the end of each walk, volunteers completed an exit questionnaire (Table 1) containing 13 Likert-scale questions about their perspectives of the walk. The 13 questions were classified into human satisfaction factor (factor H) and walkers' perception of dog factor (factor D). A higher score for factor H indicated that the handler was more satisfied with the interaction; a higher score for factor D indicated that the handler considered the dog better behaved, more supportive and being helped by the handler (Hao-Yu 39). Please refer to Shih et al. (41) for more details about the study design and survey instruments.

**Table 1:** Exit questionnaire for volunteers (*n* = 74) following walking dogs (*n* = 111) on a designated route at RSPCA Queensland, requiring them to rate each question on a 5-point scale from 1 (strongly disagree) to 5 (strongly agree) (41).

1. The dog's behaviour was good
2. I could not handle the dog well
3. I felt comfortable when interacting with the dog
4. I was physically tense
5. Overall, this is a good experience
6. The interaction was challenging for me
7. The dog did not understand me well
8. I did not feel that I was helping the dog
9. I felt supported by the dog
10. I did not enjoy its company
11. I would love to walk this dog again on another day
12. I don't think this dog is suitable for a non-experienced adopter
13. I think the dog is ready for adoption

*Human satisfaction factor (Factor H): 2, 3, 4, 5, 6, 10, 11. Walker's perception of dog factor (Factor D): 1, 7, 8, 9, 12, 13. Factor loadings for the 13 items in the exit questionnaire can be found in Shih et al. (41). Statements 2, 4, 6, 7, 8, 10, and 12 require reverse scoring*.

### Data Analysis

#### Video Recordings of Dog and Human Behaviour

Videos were coded in their entirety with Boris© behaviour observation software (46) using a continuous recording method. Canine behaviours (Table 2), human verbal cues (Table 3) and human body language (Table 4) were coded using ethograms developed based on previous research (12, 15, 27, 47–49) and modified during practise sessions. These tables are reproduced from Shih et al. (41) to aid understanding of this paper. To blind the coder, video coding was completed prior to any analysis of demographics. Twenty percent of the videos were double-coded to check intra-rater reliability (average Cohen's Kappa = 0.89). More information related to video analysis was described in Shih et al. (41).

**Table 2:** Ethogram for canine behaviour.

**Behaviour**	**Description**	**Behaviour type**	**References**
Track	Dog moves along the ground with head lowered, using nose to follow a scent	State event	(47)
Sniff	Dog orientates nose to an object, wall or ground to explore or to express stress or appeasement	State event	(47)
Eliminate-mark	Dog defecates or urinates in sitting, squatting or standing position	Point event	(48)
Shake	Dog shakes its body or head	Point event	
Pant	Dog keeps its mouth wide open and breathes vigorously	State event	(47)
Gaze	Dog looks towards the handler	Point event	(47)
Lip-lick	Part of tongue is shown and moved along the upper lip or snout	Point event	(47)
Tail wag	Tail is moving from side to side	State event	(27)
Tail high	Tail is held stiffly and upright, either curled over the back or straight	State event	(49)

*Point event: the number of times the event was observed. State event: the duration of the observed event*.

**Table 3:** Ethogram for human verbal cues.

**Behaviour**	**Description**	**Behaviour type**	**References**
Sit	Volunteer asks the dog to sit.	Point event	
Command	Volunteer talks to the dog with an utterance containing a single command (e.g., “Stay!” “Come!” “Let's go!”)	Point event	(12)
Attention seeking	Volunteer tries to get the attention of the dog and calls the dog by its name and/or the utterance of “Look!” and/or clicking the tongue (“tze tze” sound)	Point event	(12)
High-pitched voice	Volunteer talks to the dog with high pitched voice or with baby-talk expressions	Point event	(27)
Praise	Volunteer talks to the dog with a positive utterance (e.g., “Great!” “Well done!” “Good dog!”)	Point event	(12, 27)
Negative verbal cue	Volunteer talks to the dog with a negative utterance [e.g., “No!” “Bad dog!” “Don't …” “Stop chewing the lead” “Let the lead (it) go”]	Point event	
Communication	Volunteer tries to communicate with the dog or to ask the dog some questions. (e.g., “Which way do you want to go?” “What are you sniffing at?” “Do you want to fetch?” “Do you want to drink?”)	Point event	(15)

*Point event: the number of times the event was observed. State event: the duration of the observed event*.

**Table 4:** Ethogram of human body language.

**Behaviour**	**Description**	**Behaviour type**	**References**
Gestural	Volunteers displays voluntary hand movement directed towards the dog (e.g., referential point, patting his/her own thigh, luring the dog with a hand or food)	Point event	(12, 27)
Physical contacts	Physical contacts initiated by the volunteer. Including contacts when treats were given	Point event	
Food reward	Food is given to the dog	Point event	

*Point event: the number of times the event was observed*.

#### Leash Tension and Pulling Frequency

Leash tension and pulling directions were calculated using MATLAB® (MATLAB® and Statistics Toolbox Release 2018b, The MathWorks, Inc., Natick, MA, USA). A “pull event” was defined as a sharp peak of tension, which corresponded to a sudden burst of pulling initiated by either the dog, the handler or both at the same time. Events started when the tension exceeded a threshold (0.1% of the body weight force) and ended when either the tension returned to below the threshold or the direction of the pulling changed (39).

Net maximal tension (NTmax), maximal tension by dog (DTmax) and handler (HTmax) were defined as the maximal tension throughout the walk, caused by the dog and handler, respectively. Net mean tension (NTmean), mean tension by dog (DTmean) and handler (HTmean) were defined as the mean tension throughout the walk, caused by the dog and handler, respectively. Dog pulling frequency (DPF) and handler pulling frequency (HPF) were calculated by dividing the number of pulling events recorded for the dog and the handler, respectively, by the total walking time (39).

### Statistical Analysis

Statistical analysis was conducted using RStudio Version 1.2.1335 (50) with packages leaps (51), MASS (52), car (53), carData (54), Matrix (55), polycor (56), plyr (57), psych (58), ggpubr (59), and nlme (60). The same statistical analysis methods as described in a previous paper (41) were followed in this study. Bivariate generalised linear models were used to analyse each combination of outcome (leash tension, behaviour and exit questionnaire score) and predictor (human and dog demographics, human personality, canine behavioural assessment) variables, followed by generalised linear mixed models for multivariable analyses and repetitions of dogs and volunteers. Predictors with *p*-values lower than 0.2 (61, 62) in bivariate generalised linear models and those (e.g., human personality and canine behavioural assessment results) logically expected to affect the outcome variable, regardless of the *p*-value, were included in the generalised linear mixed model. Outcome variables were transformed for statistical analysis to meet the assumptions of generalised linear mixed models, including the normality of residual and random effects, homogeneity of variance of residuals and the assumption of no collinearity between covariates was confirmed from variance inflation factors (VIF, ensuring that VIF <2) (41, 63).

This article is a part of a larger research project investigating the behavioural interaction between shelter dogs and volunteers during on-leash walks. This paper focuses on correlations between human demographics and behavioural interactions. The effect of human gender (41) and personality (44) have been reported in previous articles. The effects of canine factors (39, 41) and canine behavioural assessment results (43) have also been reported.

## Results

### Demographics

This study involved 111 shelter dogs including 58 (52.3%) females and 53 (47.7%) males, all gonadectomized (41). Participants were 47 (63.5%) women, 26 (35.1%) men and 1 (1.4%) person self-nominating as a third gender (41), with an average age of 28.26 (± 14.6) years. Volunteers' average body height was 170.7 (±8.8) cm. Volunteers' educational levels were as follows: 2 (2.7%) primary school, 38 (51.4%) secondary school, 16 (21.6%) training college, 12 (16.2%) bachelor and 6 (8.1%) postgraduate. There were 49 (66.2%) participants who were single, 21 (28.4%) married/partnered and 4 (5.4%) separated/divorced or widowed. There were 21 (28.4%) level 2 volunteers, 15 (20.3%) level 3 volunteers and 38 (51.4%) level 3+ volunteers. Twenty-five (33.8%) participants volunteered more than once a week, 48 (64.9%) volunteered once a week and 1 (1.4%) volunteered fortnightly. Twelve participants (16.2%) had volunteering experience at the RSPCA that was longer than 2 years, 19 (25.7%) had 1–2 years, 23 (31.1%) had 6–12 months, 19 (25.7) had 1–6 months and 1 (1.4%) had less than a month. Fifty (67.6%) volunteers had, and 24 (32.4%) did not have, dogs of their own at the time of the research. Seventy (94.6%) volunteers had lived with dogs previously, while 4 (5.4%) had not. Fifty-nine (79.7%) volunteers grew up with dogs in the household while 15 (20.3%) did not. Seventeen (23%) volunteers had a child/children living in the household while 57 (77%) did not. Twenty (27%) volunteers worked in an area that dealt with dogs regularly while 54 (73%) did not.

### Human Demographics and Leash Tension

Volunteers' height was positively correlated with pulling frequency created by the dog (*p* < 0.001). Compared to level 3+ volunteers, level 2 volunteers had lower net mean tension (*p* = 0.035). When walking with volunteers who had lived with dogs before, dogs created lower pulling frequency (*p* = 0.0023). Lower net maximal tension (*p* = 0.0025) and maximal tension by dogs (*p* = 0.0023) were observed when volunteers reported growing up with dogs (Table 5).

**Table 5:** Generalised linear mixed model of the effects of human demographics on the leash tension and pulling frequency during the walk.

	**Log_**10**_NT_**max**_**	**Log_**10**_NT_**mean**_**	**Log_**10**_DT_**max**_**	**Log_**10**_DT_**mean**_**	**DPF^**a**^**	**Log_**10**_HT_**max**_**	**Log_**10**_HT_**mean**_**	**HPF^**a**^**
2. Age	–	β 0.001 SE 0.0019 *p* 0.58	–	β 0.0014 SE 0.0018 *p* 0.43	β −0.005 SE 0.0032 *p* 0.12	–	–	β −0.00016 SE 0.0029 *p* 0.95
3. Height	–	–	–	–	β 0.021 SE 0.005 *p* <0.001	–	–	–
5. Educational level	Postgraduate μ 3.65, SD 2.16 –	Postgraduate μ 0.69, SD 0.31 –	Postgraduate μ 3.2, SD 1.82 –	Postgraduate μ 1.25, SD 0.51 –	Postgraduate μ 0.19, SD 0.13 –	Postgraduate μ 3.1, SD 1.91 –	Postgraduate μ 1.25, SD 0.59 –	Postgraduate μ 0.19, SD 0.12 –
	Bachelor μ 3.49, SD 1.96 –	Bachelor μ 0.56, SD 0.23 –	Bachelor μ 3.08, SD 1.91 –	Bachelor μ 1.09, SD 0.44 –	Bachelor μ 0.17, SD 0.12 –	Bachelor μ 2.78, SD 1.6 –	Bachelor μ 1.06, SD 0.41 –	Bachelor μ 0.18, SD 0.12 –
	Training college μ 3.53, SD 1.93 –	Training college μ 0.57, SD 0.28 –	Training college μ 2.93, SD 1.67 –	Training college μ 1.09, SD 0.49 –	Training college μ 0.16, SD 0.12 –	Training college μ 2.86, SD 1.65 –	Training college μ 1.09, SD 0.48 –	Training college μ 0.16, SD 0.11 –
	Secondary school μ 3.84, SD 1.99	Secondary school μ 0.58, SD 0.22	Secondary school μ 3.35, SD 1.83	Secondary school μ 1.18, SD 0.48	Secondary school μ 0.2, SD 0.14	Secondary school μ 3.2, SD 1.77	Secondary school μ 1.18, SD 0.51	Secondary school μ 0.19, SD 0.13
	Primary school μ 3.66, SD 2.07 –	Primary school μ 0.7, SD 0.38 –	Primary school μ 3.34, SD 1.95 –	Primary school μ 1.31, SD 0.76 –	Primary school μ 0.2, SD 0.16 –	Primary school μ 3.3, SD 2.21 –	Primary school μ 1.21, SD 0.71 –	Primary school μ 0.17, SD 0.1 –
6. Relationship status	Married/partnered μ 3.52, SD 1.98 –	Married/partnered μ 0.57, SD 0.28 –	Married/partnered μ 3.18, SD 1.98 –	Married/partnered μ 1.13, SD 0.53 β −0.02 SE 0.043 *p* 0.64	Married/partnered μ 0.17, SD 0.12 –	Married/partnered μ 2.83, SD 1.51 –	Married/partnered μ 1.1, SD 0.5 –	Married/partnered μ 0.19, SD 0.14 –
	Separated/divorced or widowed μ 3.36, SD 1.38 –	Separated/divorced or widowed μ 0.52, SD 0.23 –	Separated/divorced or widowed μ 2.99, SD 1.48 –	Separated/divorced or widowed μ 1.03, SD 0.34 β −0.18 SE 0.1 *p* 0.077	Separated/divorced or widowed μ 0.13, SD 0.13 –	Separated/divorced or widowed μ 2.7, SD 1.13 –	Separated/divorced or widowed μ 1.06, SD 0.38 –	Separated/divorced or widowed μ 0.18, SD 0.13 –
	Single μ 3.8, SD 2.03	Single μ 0.6, SD 0.23	Single μ 3.23, SD 1.76	Single μ 1.18, SD 0.48	Single μ 0.2, SD 0.14	Single μ 3.18, SD 1.86	Single μ 1.17, SD 0.51	Single μ 0.18, SD 0.12
7. Training level	Level 2 μ 3.42, SD 1.83 β −0.011 SE 0.066 *p* 0.87	Level 2 μ 0.52, SD 0.22 β −0.13 SE 0.061 *p* 0.035	Level 2 μ 3.02, SD 1.79 β −0.021 SE 0.071 *p* 0.77	Level 2 μ 1.05, SD 0.41 β −0.061 SE 0.051 *p* 0.23	Level 2 μ 0.16, SD 0.13 β −0.044 SE 0.1 *p* 0.67	Level 2 μ 2.7, SD 1.4 β 0.0017 SE 0.071 *p* 0.98	Level 2 μ 1.03, SD 0.41 β −0.077 SE 0.05 *p* 0.12	Level 2 μ 0.16, SD 0.12 β −0.08 SE 0.091 *p* 0.38
	Level 3 μ 3.76, SD 2.22 β −0.07 SE 0.066 *p* 0.29	Level 3 μ 0.62, SD 0.26 β 0.015 SE 0.062 *p* 0.81	Level 3 μ 3.19, SD 1.95 β −0.11 SE 0.072 *p* 0.12	Level 3 μ 1.19, SD 0.53 β −0.018 SE 0.053 *p* 0.73	Level 3 μ 0.18, SD 0.13 β −0.089 SE 0.11 *p* 0.4	Level 3 μ 3.35, SD 1.99 β 0.046 SE 0.071 *p* 0.52	Level 3 μ 1.21, SD 0.54 β 0.02 SE 0.049 *p* 0.69	Level 3 μ 0.19, SD 0.14 β 0.044 SE 0.092 *p* 0.63
	Level 3+ μ 3.82, SD 1.96	Level 3+ μ 0.61, SD 0.25	Level 3+ μ 3.31, SD 1.77	Level 3+ μ 1.2, SD 0.5	Level 3+ μ 0.2, SD 0.14	Level 3+ μ 3.13, SD 1.78	Level 3+ μ 1.19, SD 0.52	Level 3+ μ 0.19, SD 0.12
8. Volunteering frequency	>Once a week μ 3.65, SD 1.82	>Once a week μ 0.58, SD 0.2	>Once a week μ 3.21, SD 1.74	>Once a week μ 1.14, SD 0.43	>Once a week μ 0.18, SD 0.12	>Once a week μ 2.98, SD 1.56	>Once a week μ 1.12, SD 0.48	>Once a week μ 0.16, SD 0.1
	Once a week μ 3.7, SD 2.07 –	Once a week μ 0.59, SD 0.27 –	Once a week μ 3.18, SD 1.85 –	Once a week μ 1.16, SD 0.51 –	Once a week μ 0.19, SD 0.14 –	Once a week μ 3.07, SD 1.84 –	Once a week μ 1.16, SD 0.51 –	Once a week μ 0.19, SD 0.14 –
9. Volunteering time	> 2 years μ 3.93, SD 2.06 –	> 2 years μ 0.67, SD 0.3 –	> 2 years μ 3.53, SD 2.02 –	> 2 years μ 1.24, SD 0.57 –	> 2 years μ 0.2, SD 0.13 –	> 2 years μ 3.08, SD 1.81 –	> 2 years μ 1.18, SD 0.51 –	> 2 years μ 0.2, SD 0.13 –
	1–2 years μ 3.79, SD 2.05 –	1–2 years μ 0.59, SD 0.23 –	1–2 years μ 3.26, SD 1.81 –	1–2 years μ 1.18, SD 0.51 –	1–2 years μ 0.18, SD 0.13 –	1–2 years μ 3.14, SD 1.93 –	1–2 years μ 1.17, SD 0.57 –	1–2 years μ 0.17, SD 0.12 –
	6–12 months μ 3.35, SD 1.71	6–12 months μ 0.54, SD 0.24	6–12 months μ 2.92, SD 1.7	6–12 months μ 1.06, SD 0.42	6–12 months μ 0.17, SD 0.12	6–12 months μ 2.86, SD 1.4	6–12 months μ 1.08, SD 0.44	6–12 months μ 0.17, SD 0.11
	1–6 months μ 3.88, SD 2.17 –	1–6 months μ 0.58, SD 0.24 –	1–6 months μ 3.27, SD 1.81 –	1–6 months μ 1.2, SD 0.49 –	1–6 months μ 0.2, SD 0.15 –	1–6 months μ 3.16, SD 1.88 –	1–6 months μ 1.17, SD 0.5 –	1–6 months μ 0.21, SD 0.15 –
10. Living with dog	Yes μ 3.66, SD 1.92 –	Yes μ 0.57, SD 0.24 β −0.081 SE 0.053 *p* 0.13	Yes μ 3.18, SD 1.76 –	Yes μ 1.15, SD 0.49 –	Yes μ 0.19, SD 0.14 –	Yes μ 3.05, SD 1.72 –	Yes μ 1.15, SD 0.52 –	Yes μ 0.19, SD 0.13 –
	No μ 3.77, SD 2.12	No μ 0.62, SD 0.27	No μ 3.24, SD 1.93	No μ 1.17, SD 0.48	No μ 0.17, SD 0.12	No μ 3.05, SD 1.78	No μ 1.14, SD 0.47	No μ 0.17, SD 0.12
11. Lived with dog	Yes μ 3.69, SD 1.99 –	Yes μ 0.58, SD 0.24 β −0.076 SE 0.11 *p* 0.49	Yes μ 3.19, SD 1.8 –	Yes μ 1.15, SD 0.48 β −0.11 SE 0.083 *p* 0.17	Yes μ 0.18, SD 0.13 β −0.51 SE 0.17 *p* 0.0023	Yes μ 3.05, SD 1.75 –	Yes μ 1.14, SD 0.5 –	Yes μ 0.18, SD 0.13 –
	No μ 3.8, SD 1.99	No μ 0.68, SD 0.33	No μ 3.47, SD 2.05	No μ 1.27, SD 0.59	No μ 0.2, SD 0.12	No μ 3.04, SD 1.66	No μ 1.18, SD 0.53	No μ 0.19, SD 0.1
12. Grew up with dog	Yes μ 3.61, SD 1.88 β −0.19 SE 0.064 *p* 0.0025	Yes μ 0.58, SD 0.23 –	Yes μ 3.13, SD 1.75 β −0.21 SE 0.068 *p* 0.0023	Yes μ 1.15, SD 0.46 –	Yes μ 0.19, SD 0.14 –	Yes μ 3.01, SD 1.61 β −0.12 SE 0.069 *p* 0.072	Yes μ 1.14, SD 0.48 –	Yes μ 0.18, SD 0.13 –
	No μ 4.01, SD 2.33	No μ 0.59, SD 0.31	No μ 3.47, SD 2.02	No μ 1.2, SD 0.6	No μ 0.17, SD 0.1	No μ 3.21, SD 2.19	No μ 1.17, SD 0.59	No μ 0.17, SD 0.1
13. Child/children at home	Yes μ 3.86, SD 2.07 –	Yes μ 0.61, SD 0.21 β 0.049 SE 0.061 *p* 0.42	Yes μ 3.32, SD 1.6 β −0.06 SE 0.069 *p* 0.39	Yes μ 1.22, SD 0.51 β 0.0041 SE 0.051 *p* 0.94	Yes μ 0.2, SD 0.15 β 0.033 SE 0.1 *p* 0.75	Yes μ 3.24, SD 2.03 –	Yes μ 1.21, SD 0.59 –	Yes μ 0.21, SD 0.14 β 0.11 SE 0.093 *p* 0.25
	No μ 3.64, SD 1.96	No μ 0.58, SD 0.26	No μ 3.17, SD 1.87	No μ 1.14, SD 0.48	No μ 0.18, SD 0.13	No μ 3, SD 1.64	No μ 1.13, SD 0.47	No μ 0.17, SD 0.12
14. Work with dogs	Yes μ 3.75, SD 1.9 –	Yes μ 0.61, SD 0.27 –	Yes μ 3.33, SD 1.93 –	Yes μ 1.19, SD 0.56 –	Yes μ 0.19, SD 0.13 –	Yes μ 3.11, SD 1.72 –	Yes μ 1.15, SD 0.56 –	Yes μ 0.19, SD 0.13 –
	No μ 3.67, SD 2.02	No μ 0.58, SD 0.24	No μ 3.16, SD 1.77	No μ 1.15, SD 0.46	No μ 0.18, SD 0.13	No μ 3.03, SD 1.75	No μ 1.14, SD 0.48	No μ 0.18, SD 0.12

*Question 1, human gender, was reported in the previous study (41). Question 4 is a repetitive question of questions 2 and 9; also, high VIF values (VIF>2) were detected, so question 4 was excluded from the analysis.*

*Tension and pulling frequency were analysed in log_10_ transformation. NT_max_, maximal net leash tension; NT_mean_, mean net leash tension; DT_max_, maximal leash tension caused by dog; DT_mean_, mean leash tension caused by dog; HT_max_, maximal leash tension caused by handler; HT_mean_, mean leash tension caused by handler; DPF, dog pulling frequency; HPF, handler pulling frequency.*

a *Pulling frequency = (Numbers of pulls)/(walking duration).*

*A pull was defined as a bout of force >0.1% of the dog's body weight force. Question 5, educational level, secondary school was used for comparison. Question 6, relationship status, volunteers who were singled were used for comparison. Question 7, training level, level 3+ was used for comparison. Question 8, volunteering frequency, frequency greater than once a week was used for comparison. Only one participant volunteered fortnightly. To prevent bias, this participant was excluded from the analysis related to volunteering frequency. Question 9, volunteering time between 6 and 12 months was used for comparison. Only 1 volunteer had volunteering experience <1 month. To prevent bias, the person was excluded from analysis of volunteering time. Question 10, volunteers who was not living with a dog at the time of conducting the research were used for comparison. Question 11, volunteers who had not lived with a dog before the time of conducting the research were used for comparison. Question 12, volunteers who did not grow up with a dog were used for comparison. Question 13, volunteers who did not live with a child/children at the time of conducting the research were used for comparison. Question 14, volunteers who did not work with a dog at the time of conducting the research were used for comparison. μ: mean (before transformation) (kg force). SD: standard deviation of μ. β: regression coefficient. SE: standard error of β. p: p value of the model. –: Not included in the generalised linear mixed model because the predictor had high p-values in the bivariate regression model*.

### Human Demographics and Human Behaviour

Volunteers' age was positively correlated with frequency of total verbal cues (*p* < 0.001), communication (*p* = 0.033), praise (*p* = 0.043), and command (*p* < 0.001). Volunteers' height was positively related to frequencies of high-pitched voice (*p* = 0.02) and command (*p* = 0.023) but negatively associated with the frequency of communication (*p* < 0.001). Compared to secondary school, volunteers holding a postgraduate degree used a higher frequency of total verbal cue (*p* = 0.042); volunteers with bachelor (*p* = 0.0022), training college (*p* = 0.0083) and primary school (*p* = 0.032) as their highest educational levels used a lower frequency of total verbal cue. Specifically, holding a postgraduate degree was positively associated with the frequency of praise (*p* = 0.0022); a bachelor's degree was negatively associated with frequencies of communication (*p* = 0.016) and command (*p* = 0.0012); training college was negatively associated with the frequency of command (*p* < 0.001); primary school was negatively associated with the frequency of praise (*p* < 0.001). Compared to volunteers who were single, married/partnered volunteers used higher frequencies of total verbal cue (*p* = 0.014) and praise (*p* = 0.0034).

Compared to level 3+ volunteers, level 3 volunteers used higher frequencies of total verbal cue (*p* < 0.001), communication (*p* = 0.033), praise (*p* < 0.001), high-pitched voice (*p* = 0.0013) and command (*p* < 0.001). Level 2 volunteers used a higher frequency of command (*p* = 0.011). Compared to volunteers who came more frequently than once a week, those who came once a week used a lower frequency of attention seeking commands (*p* = 0.023), but a higher frequency of praise (*p* = 0.043).

Volunteers who were living with dogs at the time of conduction of the experiment and those who grew up with dogs used lower frequencies of high-pitched voice (*p* = 0.015) and total verbal cues (*p* = 0.017), respectively. Volunteers working with dogs used higher frequencies of attention seeking (*p* = 0.035), total verbal cues (*p* < 0.001), praise (*p* = 0.0074) and verbs (*p* < 0.001) (Table 6).

**Table 6:** Generalised linear mixed model of the effects of human demographics on human verbal cues during the walk.

	**Total verbal cues (no./sec)^**a**^**	**Attention seeking (no./sec)^**b**^**	**Communication (no./sec)^**b**^**	**Negative verbal cue (no./sec)^**b**^**	**Praise (no./sec)^**a**^**	**High-pitched voice (no./sec)^**a**^**	**Command (no./sec)^**a**^**
2. Age	β 0.0023 SE 0.00068 *p* <0.001	β 0.00087 SE 0.00065 *p* 0.18	β 0.001 SE 0.00047 *p* 0.033	–	β 0.0097 SE 0.00048 *p* 0.043	β 0.00049 SE 0.0004 *p* 0.23	β 0.0019 SE 0.00046 *p* <0.001
3. Height	β 0.001 SE 0.00086 *p* 0.23	β 0.001 SE 0.00086 *p* 0.23	β −0.0018 SE 0.00055 *p* <0.001	–	–	β 0.0013 SE 0.00056 *p* 0.02	β 0.0014 SE 0.0006 *p* 0.023
5. Educational level	Postgraduate μ 0.11, SD 0.07 β 0.059 SE 0.029 *p* 0.042	Postgraduate μ 0.02, SD 0.03 –	Postgraduate median <0.01 IQR 0.01 β −0.014 SE 0.018 *p* 0.41	Postgraduate median <0.01 IQR <0.01 –	Postgraduate μ 0.04, SD 0.03 β 0.064 SE 0.021 *p* 0.0022	Postgraduate median <0.01 IQR 0.02 –	Postgraduate μ 0.04, SD 0.03 β 0.029 SE 0.02 *p* 0.15
	Bachelor μ 0.08, SD 0.07 β −0.066 SE 0.021 *p* 0.0022	Bachelor μ 0.02, SD 0.02 –	Bachelor median <0.01 IQR 0.01 β −0.035 SE 0.015 *p* 0.016	Bachelor median <0.01 IQR <0.01 –	Bachelor μ 0.03, SD 0.04 β −0.02 SE 0.015 *p* 0.19	Bachelor median <0.01 IQR 0.02 –	Bachelor μ 0.03, SD 0.03 β −0.049 SE 0.015 *p* 0.0012
	Training college μ 0.08, SD 0.06 β −0.048 SE 0.018 *p* 0.0083	Training college μ 0.02, SD 0.02 –	Training college median <0.01 IQR 0.01 β 0.00017 SE 0.013 *p* 0.99	Training college median <0.01 IQR <0.01 –	Training college μ 0.02, SD 0.02 β −0.0011 SE 0.014 *p* 0.94	Training college median <0.01 IQR 0.01 –	Training college μ 0.03, SD 0.03 β −0.049 SE 0.013 *p* <0.001
	Secondary school μ 0.08, SD 0.06	Secondary school μ 0.02, SD 0.02	Secondary school median <0.01 IQR <0.01	Secondary school median <0.01 IQR <0.01	Secondary school μ 0.02, SD 0.02	Secondary school median <0.01 IQR 0.02	Secondary school μ 0.04, SD 0.03
	Primary school μ 0.05, SD 0.03 β −0.096 SE 0.045 *p* 0.032	Primary school μ 0.01, SD 0.01 –	Primary school median <0.01 IQR 0.01 β −0.033 SE 0.032 *p* 0.31	Primary school median <0.01 IQR <0.01 –	Primary school μ <0.01, SD 0.01 β −0.147 SE 0.033 *p* <0.001	Primary school median 0.01 IQR 0.02 –	Primary school μ 0.03, SD 0.02 β −0.033 SE 0.031 *p* 0.28
6. Relationship status	Married/partnered μ 0.1, SD 0.07 β 0.039 SE 0.016 *p* 0.014	Married/partnered μ 0.02, SD 0.02 β 0.028 SE 0.017 *p* 0.097	Married/partnered median <0.01 IQR 0.01 β 0.02 SE 0.011 *p* 0.07	Married/partnered median <0.01 IQR <0.01 β −0.007 SE 0.0085 *p* 0.41	Married/partnered μ 0.03, SD 0.03 β 0.035 SE 0.012 *p* 0.0034	Married/partnered median <0.01 IQR 0.02 β 0.0067 SE 0.011 *p* 0.53	Married/partnered μ 0.03, SD 0.03 β 0.016 SE 0.011 *p* 0.14
	Separated/divorced or widowed μ 0.08, SD 0.06 β −0.015 SE 0.041 *p* 0.72	Separated/divorced or widowed μ 0.01, SD 0.01 β −0.048 SE 0.04 *p* 0.23	Separated/divorced or widowed median <0.01 IQR 0.01 β −0.0055 SE 0.026 *p* 0.83	Separated/divorced or widowed median <0.01 IQR <0.01 β 0.041 SE 0.021 *p* 0.058	Separated/divorced or widowed μ 0.03, SD 0.03 β −0.033 SE 0.028 *p* 0.24	Separated/divorced or widowed median <0.01 IQR <0.01 β −0.032 SE 0.027 *p* 0.25	Separated/divorced or widowed μ 0.04, SD 0.04 β −0.031 SE 0.028 *p* 0.27
	Single μ 0.07, SD 0.07	Single μ 0.02, SD 0.02	Single median <0.01 IQR <0.01	Single median <0.01 IQR <0.01	Single μ 0.02, SD 0.02	Single median <0.01 IQR 0.02	Single μ 0.03, SD 0.03
7. Training level	Level 2 μ 0.08, SD 0.06 β 0.015 SE 0.016 *p* 0.36	Level 2 μ 0.02, SD 0.02 β −0.014 SE 0.018 *p* 0.43	Level 2 median <0.01 IQR 0.01 β 0.0017 SE 0.0123 *p* 0.9	Level 2 median <0.01 IQR <0.01 β 0.00011 SE 0.0098 *p* 0.99	Level 2 μ 0.02, SD 0.02 β 0.00078 SE 0.012 *p* 0.95	Level 2 median <0.01 IQR 0.02 β 0.017 SE 0.011 *p* 0.13	Level 2 μ 0.04, SD 0.03 β 0.029 SE 0.011 *p* 0.011
	Level 3 μ 0.1, SD 0.07 β 0.066 SE 0.018 *p* <0.001	Level 3 μ 0.02, SD 0.02 β 0.028 SE 0.019 *p* 0.13	Level 3 median <0.01 IQR 0.01 β 0.029 SE 0.013 *p* 0.033	Level 3 median <0.01 IQR <0.01 β 0.0077 SE 0.0097 *p* 0.43	Level 3 μ 0.03, SD 0.03 β 0.051 SE 0.013 *p* <0.001	Level 3 median 0.01 IQR 0.02 β 0.038 SE 0.012 *p* 0.0013	Level 3 μ 0.04, SD 0.04 β 0.048 SE 0.013 *p* <0.001
	Level 3+ μ 0.08, SD 0.06	Level 3+ μ 0.02, SD 0.02	Level 3+ median <0.01 IQR <0.01	Level 3+ median <0.01 IQR <0.01	Level 3+ μ 0.02, SD 0.02	Level 3+ median <0.01 IQR 0.01	Level 3+ μ 0.03, SD 0.03
8. Volunteering frequency	>Once a week μ 0.08, SD 0.07	>Once a week μ 0.02, SD 0.03	>Once a week median <0.01 IQR <0.01	>Once a week median <0.01 IQR <0.01	>Once a week μ <0.01, SD <0.01	>Once a week median <0.01 IQR 0.01	>Once a week μ 0.03, SD 0.03
	Once a week μ 0.08, SD 0.07 –	Once a week μ 0.02, SD 0.02 β −0.032 SE 0.014 *p* 0.023	Once a week median <0.01 IQR 0.01 β 0.011 SE 0.01 *p* 0.29	Once a week median <0.01 IQR <0.01 –	Once a week μ <0.01, SD <0.01 β 0.023 SE 0.011 *p* 0.043	Once a week median <0.01 IQR 0.02 β −0.0011 SE 0.0093 *p* 0.9	Once a week μ 0.03, SD 0.03 –
9. Volunteering time	>2 years μ 0.1, SD 0.08 –	>2 years μ 0.02, SD 0.03 β −0.024 SE 0.022 *p* 0.27	>2 years median <0.01 IQR 0.01 β −0.014 SE 0.014 *p* 0.32	>2 years median <0.01 IQR <0.01 –	>2 years μ <0.01, SD <0.01 –	>2 years median <0.01 IQR <0.01 –	>2 years μ 0.03, SD 0.04 –
	1–2 years μ 0.07, SD 0.06 –	1–2 years μ 0.02, SD 0.03 β −0.028 SE 0.019 *p* 0.14	1–2 years median <0.01 IQR <0.01 β −0.016 SE 0.012 *p* 0.2	1–2 years median <0.01 IQR <0.01 –	1–2 years μ <0.01, SD 0.01 –	1–2 years median <0.01 IQR 0.01 –	1–2 years μ 0.02, SD 0.03 –
	6–12 months μ 0.09, SD 0.06	6–12 months μ 0.02, SD 0.02	6–12 months median <0.01 IQR 0.01	6–12 months median <0.01 IQR <0.01	6–12 months μ <0.01, SD 0.01	6–12 months median 0.01 IQR 0.02	6–12 months μ 0.03, SD 0.03
	1–6 months μ 0.08, SD 0.07 –	1–6 months μ 0.01, SD 0.02 β −0.054 SE 0.017 *p* 0.0016	1–6 months median <0.01 IQR 0.01 β −0.016 SE 0.011 *p* 0.15	1–6 months median <0.01 IQR <0.01 –	1–6 months μ <0.01, SD <0.01 –	1–6 months median 0.01 IQR 0.02 –	1–6 months μ 0.04, SD 0.03 –
10. Living with dog	Yes μ 0.08, SD 0.06 –	Yes μ 0.02, SD 0.02 –	Yes median <0.01 IQR 0.01 –	Yes median <0.01 IQR <0.01 –	Yes μ <0.01, SD <0.01 β 0.018 SE 0.012 *p* 0.11	Yes median <0.01 IQR 0.01 β −0.026 SE 0.011 *p* 0.015	Yes μ 0.03, SD 0.03 –
	No μ 0.08, SD 0.07	No μ 0.02, SD 0.03	No median <0.01 IQR 0.01	No median <0.01 IQR <0.01	No μ <0.01, SD 0.01	No median <0.01 IQR 0.02	No μ 0.03, SD 0.03
11. Lived with dog	Yes μ 0.08, SD 0.07 –	Yes μ 0.02, SD 0.02 –	Yes median <0.01 IQR 0.01 β −0.00076 SE 0.024 *p* 0.97	Yes median <0.01 IQR <0.01 –	Yes μ <0.01, SD <0.01 –	Yes median <0.01 IQR 0.01 β 0.0032 SE 0.021 *p* 0.88	Yes μ 0.03, SD 0.03 β −0.04 SE 0.023 *p* 0.089
	No μ 0.08, SD 0.06	No μ 0.02, SD 0.02	No median 0.01 IQR 0.01	No median <0.01 IQR <0.01	No μ <0.01, SD <0.01	No median 0.01 IQR 0.02	No μ 0.04, SD 0.03
12. Grew up with dog	Yes μ 0.08, SD 0.07 β −0.041 SE 0.017 *p* 0.017	Yes μ 0.02, SD 0.02 –	Yes median <0.01 IQR <0.01 β −0.025 SE 0.014 *p* 0.088	Yes median <0.01 IQR <0.01 –	Yes μ <0.01, SD 0.01 β −0.028 SE 0.014 *p* 0.05	Yes median <0.01 IQR 0.01 –	Yes μ 0.03, SD 0.03 β −0.014 SE 0.013 *p* 0.29
	No μ 0.09, SD 0.06	No μ 0.02, SD 0.02	No median 0.01 IQR 0.01	No median <0.01 IQR <0.01	No μ <0.01, SD <0.01	No median <0.01 IQR 0.02	No μ 0.04, SD 0.03
13. Child/children at home	Yes μ 0.09, SD 0.07 β 0.015 SE 0.018 *p* 0.39	Yes μ 0.02, SD 0.03 –	Yes median <0.01 IQR 0.01 β 0.015 SE 0.012 *p* 0.24	Yes median <0.01 IQR <0.01 –	Yes μ <0.01, SD <0.01 –	Yes median <0.01 IQR 0.02 –	Yes μ 0.04, SD 0.04 β 0.014 SE 0.012 *p* 0.27
	No μ 0.08, SD 0.07	No μ 0.02, SD 0.02	No median <0.01 IQR <0.01	No median <0.01 IQR <0.01	No μ <0.01, SD 0.01	No median <0.01 IQR 0.02	No μ 0.03, SD 0.03
14. Work with dogs (Yes)	Yes μ 0.09, SD 0.07 β 0.056 SE 0.016 *p* <0.001	Yes μ 0.02, SD 0.03 β 0.035 SE 0.017 *p* 0.035	Yes median <0.01 IQR 0.01 –	Yes median <0.01 IQR <0.01 –	Yes μ <0.01, SD <0.01 β 0.032 SE 0.012 *p* 0.0074	Yes median <0.01 IQR 0.01 –	Yes μ 0.04, SD 0.03 β 0.052 SE 0.011 *p* <0.001
	No μ 0.08, SD 0.07	No μ 0.02, SD 0.02	No median <0.01 IQR 0.01	No median <0.01 IQR <0.01	No μ <0.01, SD 0.01	No median <0.01 IQR 0.02	No μ 0.03, SD 0.03

*Question 1, human gender, was reported in the previous study (Hao-Yu 39). Question 4 is a repetitive question of questions 2 and 9; also, high VIF values (VIF>2) were detected, so question 4 was excluded from the analysis. All verbal cues were analysed with frequency (numbers of the event/total walking time).*

a *Analysed in power of 0.5.*

b *Analysed in power of 0.4. μ: mean (before transformation).*

*SD, standard deviation of μ; IQR, interquartile range; β, regression coefficient; SE, standard error of β. p, p-value of the model. Question 5, educational level, secondary school was used for comparison. Question 6, relationship status, volunteers who were singled were used for comparison. Question 7, training level, level 3+ was used for comparison. Question 8, volunteering frequency, frequency greater than once a week was used for comparison. Only one participant volunteered fortnightly. To prevent bias, this participant was excluded from the analysis related to volunteering frequency. Question 9, volunteering time between 6 and 12 months was used for comparison. Only 1 volunteer had volunteering experience <1 month. To prevent bias, the person was excluded from analysis of volunteering time. Question 10, volunteers who was not living with a dog at the time of conducting the research were used for comparison. Question 11, volunteers who had not lived with a dog before the time of conducting the research were used for comparison. Question 12, volunteers who did not grow up with a dog were used for comparison. Question 13, volunteers who did not live with a child/children at the time of conducting the research were used for comparison. Question 14, volunteers who did not work with a dog at the time of conducting the research were used for comparison. –: Not included in the generalised linear mixed model because the predictor had high p-values in the bivariate regression model*.

With respect to human body language, volunteers' height was negatively associated with the frequency of physical contact with the dog (*p* = 0.0032). Compared to secondary school, volunteers with a postgraduate degree used higher frequencies of total body language (*p* < 0.001), food rewards (*p* = 0.0014), hand gestures (*p* = 0.0051) and physical contact (*p* < 0.001). Separated/divorced volunteers used higher frequencies of total body language (*p* = 0.045) and physical contact (*p* = 0.01) than single volunteers. Compared to level 3+ volunteers, level 3 volunteers initiated a higher frequency of physical contact (*p* = 0.037). Volunteers growing up with dogs used a lower frequency of total body language (*p* < 0.001), hand gestures (*p* < 0.001) and physical contact (*p* = 0.011). However, those having a child/children in the household used higher frequencies of total body language (*p* = 0.016), hand gestures (*p* = 0.033), and physical contact (*p* = 0.011) (Table 7).

**Table 7:** Generalised linear mixed model of the effects of human demographics on human body languages during the walk.

	**Total body language (no./sec)^**a**^**	**Food reward (no./sec)**	**Hand gesture (no./sec)^**b**^**	**Physical contact (no./sec)^**a**^**
2. Age	–	–	–	β −0.00086 SE 0.00076 *p* 0.26
3. Height	β −0.0013 SE 0.0012 *p* 0.25	–	–	β −0.0028 SE 0.00092 *p* 0.0032
5. Educational level	Postgraduate median 0.01 IQR 0.03 β 0.14 SE 0.038 *p* <0.001	Postgraduate median <0.01 IQR 0.01 β 0.0034 SE 0.0011 *p* 0.0014	Postgraduate median <0.01 IQR 0.01 β 0.062 SE 0.022 *p* 0.0051	Postgraduate median <0.01 IQR 0.01 β 0.1 SE 0.03 *p* <0.001
	Bachelor median <0.01 IQR <0.01 β −0.042 SE 0.028 *p* 0.13	Bachelor median <0.01 IQR <0.01 β −0.0014 SE 0.00086 *p* 0.11	Bachelor median <0.01 IQR <0.01 β −0.0056 SE 0.015 *p* 0.72	Bachelor median <0.01 IQR <0.01 β −0.029 SE 0.023 *p* 0.21
	Training college median <0.01 IQR 0.02 β 0.017 SE 0.024 *p* 0.49	Training college median <0.01 IQR <0.01 β −0.00051 SE 0.0008 *p* 0.52	Training college median <0.01 IQR 0.01 β 0.013 SE 0.013 *p* 0.33	Training college median <0.01 IQR 0.01 β 0.029 SE 0.021 *p* 0.16
	Secondary school median <0.01 IQR 0.01	Secondary school median <0.01 IQR <0.01	Secondary school median <0.01 IQR <0.01	Secondary school median <0.01 IQR 0.01
	Primary school median <0.01 IQR 0.01 β −0.1 SE 0.064 *p* 0.12	Primary school median <0.01 IQR <0.01 β −0.00036 SE 0.002 *p* 0.85	Primary school median <0.01 IQR <0.01 β −0.033 SE 0.035 *p* 0.35	Primary school median <0.01 IQR <0.01 β −0.085 SE 0.053 *p* 0.11
6. Relationship status	Married/partnered median <0.01 IQR 0.01 β 0.0085 SE 0.02 *p* 0.68	Married/partnered median <0.01 IQR <0.01 –	Married/partnered median <0.01 IQR <0.01 –	Married/partnered median <0.01 IQR 0.01 β 0.016 SE 0.017 *p* 0.35
	Separated/divorced or widowed median <0.01 IQR 0.01 β 0.106 SE 0.052 *p* 0.045	Separated/divorced or widowed median <0.01 IQR <0.01 –	Separated/divorced or widowed median <0.01 IQR <0.01 –	Separated/divorced or widowed median <0.01 IQR 0.01 β 0.12 SE 0.047 *p* 0.01
	Single median <0.01 IQR 0.01	Single median <0.01 IQR <0.01	Single median <0.01 IQR <0.01	Single median <0.01 IQR 0.01
7. Training level	Level 2 median <0.01 IQR 0.01 –	Level 2 median <0.01 IQR <0.01 β −0.00061 SE 0.00074 *p* 0.41	Level 2 median <0.01 IQR <0.01 –	Level 2 median <0.01 IQR <0.01 β 0.011 SE 0.018 *p* 0.56
	Level 3 median <0.01 IQR 0.01 –	Level 3 median <0.01 IQR <0.01 β −0.0007 SE 0.00083 *p* 0.4	Level 3 median <0.01 IQR 0.01 –	Level 3 median <0.01 IQR <0.01 β −0.042 SE 0.02 *p* 0.037
	Level 3+ median <0.01 IQR 0.01	Level 3+ median <0.01 IQR <0.01	Level 3+ median <0.01 IQR <0.01	Level 3+ median <0.01 IQR 0.01
8. Volunteering frequency	>Once a week median <0.01 IQR 0.01	>Once a week median <0.01 IQR <0.01	>Once a week median <0.01 IQR <0.01	>Once a week median <0.01 IQR 0.01
	Once a week median <0.01 IQR 0.01 –	Once a week median <0.01 IQR <0.01 –	Once a week median <0.01 IQR <0.01 –	Once a week median <0.01 IQR <0.01 –
9. Volunteering time	>2 years median <0.01 IQR 0.02 –	>2 years median <0.01 IQR <0.01 β −0.00032 SE 0.00086 *p* 0.71	>2 years median <0.01 IQR <0.01 –	>2 years median <0.01 IQR 0.01 –
	1–2 years median <0.01 IQR 0.01 –	1–2 years median <0.01 IQR <0.01 β 0.00062 SE 0.0008 *p* 0.44	1–2 years median <0.01 IQR 0.01 –	1–2 years median <0.01 IQR 0.01 –
	6–12 months median <0.01 IQR 0.01	6–12 months median <0.01 IQR <0.01	6–12 months median <0.01 IQR <0.01	6–12 months median <0.01 IQR 0.01
	1–6 months median <0.01 IQR <0.01 –	1–6 months median <0.01 IQR <0.01 β −0.0011 SE 0.00072 *p* 0.12	1–6 months median <0.01 IQR <0.01 –	1–6 months median <0.01 IQR <0.01 –
10. Living with dog	Yes median <0.01 IQR 0.01 –	Yes median <0.01 IQR <0.01 –	Yes median <0.01 IQR <0.01 β −0.0013 SE 0.012 *p* 0.92	Yes median <0.01 IQR 0.01 –
	No median <0.01 IQR 0.02	No median <0.01 IQR <0.01	No median <0.01 IQR 0.01	No median <0.01 IQR <0.01
11. Lived with dog	Yes median <0.01 IQR 0.01 –	Yes median <0.01 IQR <0.01 –	Yes median <0.01 IQR <0.01 –	Yes median <0.01 IQR 0.01 –
	No median <0.01 IQR 0.02	No median <0.01 IQR <0.01	No median <0.01 IQR 0.01	No median <0.01 IQR 0.01
12. Grew up with dog	Yes median <0.01 IQR 0.01 β −0.091 SE 0.025 *p* <0.001	Yes median <0.01 IQR <0.01 –	Yes median <0.01 IQR <0.01 β −0.049 SE 0.014 *p* <0.001	Yes median <0.01 IQR <0.01 β −0.052 SE 0.02 *p* 0.011
	No median 0.01 IQR 0.02	No median <0.01 IQR <0.01	No median <0.01 IQR 0.01	No median <0.01 IQR 0.01
13. Child/children at home	Yes median <0.01 IQR 0.01 β 0.06 SE 0.024 *p* 0.016	Yes median <0.01 IQR <0.01 –	Yes median <0.01 IQR 0.01 β 0.027 SE 0.013 *p* 0.033	Yes median <0.01 IQR 0.01 β 0.051 SE 0.02 *p* 0.011
	No median <0.01 IQR 0.01	No median <0.01 IQR <0.01	No median <0.01 IQR <0.01	No median <0.01 IQR <0.01
14. Work with dogs	Yes median <0.01 IQR 0.01 –	Yes median <0.01 IQR <0.01 –	Yes median <0.01 IQR <0.01 –	Yes median <0.01 IQR <0.01 –
	No median <0.01 IQR 0.01	No median <0.01 IQR <0.01	No median <0.01 IQR <0.01	No median <0.01 IQR 0.01

*Question 1, human gender, was reported in the previous study (41). Question 4 is a repetitive question of questions 2 and 9; also, high VIF values (VIF > 2) were detected, so question 4 was excluded from the analysis. All verbal cues were analysed with frequency (numbers of the event/total walking time).*

a *Analysed in power of 0.3.*

b *Analysed in power of 0.4. IQR: interquartile range.*

*β: regression coefficient. SE, standard error of β; p, p-value of the model. Question 5, educational level, secondary school was used for comparison. Question 6, relationship status, volunteers who were singled were used for comparison. Question 7, training level, level 3+ was used for comparison. Question 8, volunteering frequency, frequency greater than once a week was used for comparison. Only one participant volunteered fortnightly. To prevent bias, this participant was excluded from the analysis related to volunteering frequency. Question 9, volunteering time between 6 and 12 months was used for comparison. Only 1 volunteer had volunteering experience <1 month. To prevent bias, the person was excluded from analysis of volunteering time. Question 10, volunteers who was not living with a dog at the time of conducting the research were used for comparison. Question 11, volunteers who had not lived with a dog before the time of conducting the research were used for comparison. Question 12, volunteers who did not grow up with a dog were used for comparison. Question 13, volunteers who did not live with a child/children at the time of conducting the research were used for comparison. Question 14, volunteers who did not work with a dog at the time of conducting the research were used for comparison. –: Not included in the generalised linear mixed model because the predictor had high p-values in the bivariate regression model*.

### Human Demographics and Canine Behaviour

Volunteers' age was positively associated with the percentage of time the dog spent tracking (*p* = 0.0011) and sniffing (*p* = 0.021). Compared to when walking with volunteers with only secondary school education, dogs displayed a higher frequency of lip-licking (*p* = 0.037) when walking with volunteers with training college education, but a lower percentage of time panting (*p* <0.001) when walking with those holding a bachelor's degree as their highest educational level. A higher percentage of time spent panting was observed with dogs when walking with married/partnered volunteers than single volunteers (*p* = 0.0035). A higher percentage of time spent tail wagging was observed when walking with level 3 volunteers than level 3+ volunteers (*p* = 0.023). Compared to when walking with those volunteering more than once a week, when walking with volunteers volunteering once a week, dogs spent a lower percentage of time sniffing (*p* = 0.01). A higher frequency of shaking (*p* = 0.014) was observed when partnered with volunteers having 1–2 years of experience compared with those with 6–12 months of experience (Table 8).

**Table 8:** Generalised linear mixed model of the effects of human demographics on canine behaviours during the walk.

	**Track (%)**	**Tail high (%)^**a**^**	**Tail wag (%)^**b**^**	**Gaze (no./sec)^**c**^**	**Lip-lick (no./sec)^**c**^**	**Eliminate-mark (no./sec)^**d**^**	**Shake (no./sec)^**e**^**	**Pant (%)^**f**^**	**Sniff (%)^**f**^**
2. Age	β 0.0018 SE 0.00056 *p* 0.0011	β −0.0013 SE 0.0012 *p* 0.3	β 0.00072 SE 0.00088 *p* 0.42	–	–	β −0.00029 SE 0.00015 *p* 0.054	β 0.000046 SE 0.000029 *p* 0.12	–	β 0.0014 SE 0.0006 *p* 0.021
3. Height	–	–	–	–	–	–	β 0.000046 SE 0.000041 *p* 0.26	–	–
5. Educational level	Postgraduate μ 19.31 SD 14.31 –	Postgraduate median 88.78 IQR 35.65 –	Postgraduate median 2.17 IQR 6.25 β 0.077 SE 0.045 *p* 0.093	Postgraduate median 0.01 IQR 0.01 –	Postgraduate median 0.01 IQR 0.01 β 0.026 SE 0.02 *p* 0.19	Postgraduate median <0.01 IQR <0.01 –	Postgraduate median <0.01 IQR <0.01 –	Postgraduate μ 14.15 SD 15.59 β −0.0011 SE 0.037 *p* 0.98	Postgraduate μ 7.91, SD 5.15 –
	Bachelor μ 17.32 SD 11.63 –	Bachelor median 91.69 IQR 22.66 –	Bachelor Median <0.01 IQR 1.83 β −0.013 SE 0.035 *p* 0.72	Bachelor median 0.01 IQR 0.02 –	Bachelor median <0.01 IQR 0.01 β −0.0045 SE 0.016 *p* 0.78	Bachelor median <0.01 IQR 0.01 –	Bachelor median <0.01 IQR <0.01 –	Bachelor μ 9.38 SD 10.83 β −0.1 SE 0.028 *p* <0.001	Bachelor μ 12.18, SD 8.07 –
	Training college μ 14.59 SD 10.57 –	Training college median 89.54 IQR 30.73 –	Training college median IQR β −0.01 SE 0.031 *p* 0.74	Training college median 0.01 IQR 0.02 –	Training college median 0.01 IQR 0.02 β 0.03 SE 0.014 *p* 0.037	Training college median 0.45 IQR 0.77 –	Training college median <0.01 IQR <0.01 –	Training college μ 10.11 SD 8.86 β −0.0082 SE 0.024 *p* 0.74	Training college μ 12.57, SD 9.84 –
	Secondary school μ 13.86 SD 10.62	Secondary school median 92.97 IQR 16.13	Secondary school median 0.07 IQR 1.79	Secondary school median 0.01 IQR 0.02	Secondary school median <0.01 IQR 0.01	Secondary school median <0.01 IQR 0.01	Secondary school median <0.01 IQR <0.01	Secondary school μ 10.23 SD 11	Secondary school μ 9.58, SD 6.86
	Primary school μ 15.01 SD 12.58 –	Primary school median 90.16 IQR 22.63 –	Primary school median 0.29 IQR 5.3 β −0.055 SE 0.079 *p* 0.48	Primary school median 0.01 IQR 0.02 –	Primary school median 0.01 IQR 0.01 β 0.022 SE 0.035 *p* 0.54	Primary school median 0.01 IQR 0.01 –	Primary school median <0.01 IQR <0.01 –	Primary school μ 8.66 SD 13.37 β −0.026 SE 0.064 *p* 0.68	Primary school μ 12.4, SD 9.13 –
6. Relationship status	Married/partnered μ 14.88 SD 11.71 –	Married/partnered median 91.69 IQR 26.91 –	Married/partnered median 0.26 IQR 2.74 –	Married/partnered median 0.01 IQR 0.02 –	Married/partnered median <0.01 IQR 0.01 –	Married/partnered median <0.01 IQR 0.01 β −0.0034 SE 0.0036 *p* 0.35	Married/partnered median <0.01 IQR <0.01 –	Married/partnered μ 12.66 SD 12.71 β 0.063 SE 0.021 *p* 0.0035	Married/partnered μ 10.25, SD 8.26 –
	Separated/divorced or widowed μ 18.05 SD 13.92 –	Separated/divorced or widowed median 89.16 IQR 54.1 –	Separated/divorced or widowed median <0.01 IQR 5.12 –	Separated/divorced or widowed median 0.01 IQR 0.01 –	Separated/divorced or widowed median 0.01 IQR 0.01 –	Separated/divorced or widowed median <0.01 IQR <0.01 β 0.0074 SE 0.0085 *p* 0.39	Separated/divorced or widowed median <0.01 IQR <0.01 –	Separated/divorced or widowed μ 10.25 SD 16.41 β 0.029 SE 0.057 *p* 0.61	Separated/divorced or widowed μ 9.82, SD 6.07 –
	Single μ 14.89 SD 10.82	Single median 92.07 IQR 16.77	Single median <0.01 IQR 2.08	Single median 0.01 IQR 0.02	Single median 0.01 IQR 0.01	Single median <0.01 IQR 0.01	Single median <0.01 IQR <0.01	Single μ 9.35 SD 9.62	Single μ 10.78, SD 7.82 –
7. Training level	Level 2 μ 13.01 SD 10.26 β −0.028 SE 0.016 *p* 0.085	Level 2 median 91.46 IQR 30.23 –	Level 2 median <0.01 IQR 3.07 β −0.023 SE 0.029 *p* 0.43	Level 2 median 0.01 IQR 0.02 –	Level 2 median 0.01 IQR 0.01 –	Level 2 median <0.01 IQR 0.01 β −0.0066 SE 0.0044 *p* 0.13	Level 2 median <0.01 IQR <0.01 –	Level 2 μ 9.15 SD 11.53 β −0.0018 SE 0.024 *p* 0.94	Level 2 μ 11.58, SD 10.18 –
	Level 3 μ 13.56 SD 9.9 β −0.0022 SE 0.016 *p* 0.89	Level 3 median 91.69 IQR 18.42 –	Level 3 median <0.01 IQR 1.13 β −0.068 SE 0.03 *p* 0.023	Level 3 median 0.01 IQR 0.02 –	Level 3 median 0.01 IQR 0.01 –	Level 3 median <0.01 IQR 0.01 β −0.0061 SE 0.0045 *p* 0.18	Level 3 median <0.01 IQR <0.01 –	Level 3 μ 12.72 SD 11.51 β 0.047 SE 0.025 *p* 0.059	Level 3 μ 9.74, SD 6.16 –
	Level 3+ μ 16.78 SD 12.02	Level 3+ median 92.14 IQR 14.74	Level 3+ median 0.36 IQR 2.84	Level 3+ median 0.01 IQR 0.02	Level 3+ median <0.01 IQR 0.02	Level 3+ median <0.01 IQR 0.01	Level 3+ median <0.01 IQR <0.01	Level 3+ μ 10.06 SD 10.54	Level 3+ μ 10.37, SD 6.92
8. Volunteering frequency	>Once a week μ 14.4 SD 10.86	>Once a week median 91.96 IQR 17.54	>Once a week median 0.35 IQR 2.52	>Once a week median 0.01 IQR 0.02	>Once a week median 0.01 IQR 0.02	>Once a week median <0.01 IQR 0.01	>Once a week median <0.01 IQR <0.01	>Once a week μ 11.19 SD 12	>Once a week μ 11.13, SD 6.75
	Once a week μ 15.55 SD 11.49 –	Once a week median 91.9 IQR 19.32 –	Once a week median <0.01 IQR 2.49 –	Once a week median 0.01 IQR 0.02 –	Once a week median 0.01 IQR 0.01 β −0.0089 SE 0.012 *p* 0.46	Once a week median <0.01 IQR 0.01 β 0.0025 SE 0.0031 *p* 0.42	Once a week median <0.01 IQR <0.01 –	Once a week μ 9.98 SD 10.61 –	Once a week μ 10.24, SD 8.38 β −0.038 SE 0.015 *p* 0.01
9. Volunteering time	>2 years μ 17.13 SD 10.97 –	>2 years median 90.17 IQR 24.47 –	>2 years median 0.68 IQR 3.37 –	>2 years median 0.01 IQR 0.01 β −0.031 SE 0.016 *p* 0.057	>2 years median <0.01 IQR 0.01 –	>2 years edian <0.01 IQR 0.01 β 0.00092 SE 0.0051 *p* 0.86	>2 years median <0.01 IQR <0.01 β 0.00071 SE 0.0011 *p* 0.53	>2 years μ 8.53 SD 7.98 –	>2 years μ 11.37, SD 6.81 –
	1–2 year μ 13.24 SD 9.49 –	1–2 year median 92.72 IQR 21.8 –	1–2 year median <0.01 IQR 1.94 –	1–2 years median 0.01 IQR 0.02 β −0.0084 SE 0.015 *p* 0.58	1–2 year median 0.01 IQR 0.02 –	1–2 year median <0.01 IQR 0.01 β −0.0021 SE 0.0041 *p* 0.61	1–2 year median <0.01 IQR <0.01 β −0.0025 SE 0.00099 *p* 0.014	1–2 year μ 10.46 SD 9.85 –	1–2 year μ 10.72, SD 7.11 –
	6–12 months μ 15.42 SD 11.99	6–12 months median 90.45 IQR 14.49	6–12 months median 0.44 IQR 2.53	6–12 months median 0.01 IQR 0.02	6–12 months median 0.01 IQR 0.02	6–12 months median <0.01 IQR 0.01	6–12 months median <0.01 IQR <0.01	6–12 months μ 9.36 SD 10.29	6–12 months μ 11.55, SD 9.58
	1–6 months μ 15.31 SD 12.17 –	1–6 months median 93.41 IQR 20.89 –	1–6 months median 0.34 IQR 2.13 –	1–6 months median 0.01 IQR 0.02 β −0.028 SE 0.014 *p* 0.055	1–6 months median <0.01 IQR 0.01 –	1–6 months median <0.01 IQR 0.01 β −0.00081 SE 0.004 *p* 0.84	1–6 months median <0.01 IQR <0.01 β −0.00065 SE 0.00093 *p* 0.48	1–6 months μ 11.75 SD 13.63 –	1–6 months μ 8.88, SD 6.73 –
10. Living with dog	Yes μ 15.01 SD 11.27 –	Yes median 92.42 IQR 16.53 β −0.0026 SE 0.033 *p* 0.94	Yes median 0.31 IQR 2.69 –	Yes median 0.01 IQR 0.02 –	Yes median 0.01 IQR 0.01 –	Yes median <0.01 IQR 0.01 β 0.0046 SE 0.0034 *p* 0.18	Yes median <0.01 IQR <0.01 –	Yes μ 10.7 SD 11.51 –	Yes μ, SD 10.05, 7.51 β −0.014 SE 0.016 *p* 0.4
	No μ 15.13 SD 11.24	No median 89.41 IQR 36.69	No median <0.01 IQR 2.05	No median 0.01 IQR 0.02	No median <0.01 IQR 0.02	No median <0.01 IQR 0.01	No median <0.01 IQR <0.01	No μ 9.6 SD 10.1	No μ 11.66, SD 8.45
11. Lived with dog	Yes μ 15.21 SD 11.47 –	Yes median 91.92 IQR 18.1 –	Yes median 0.26 IQR 2.66 β 0.0092 SE 0.057 *p* 0.87	Yes median 0.01 IQR 0.02 –	Yes median 0.01 IQR 0.01 –	Yes median <0.01 IQR 0.01 –	Yes median <0.01 IQR <0.01 –	Yes μ 10.49 SD 11.28 –	Yes μ 10.44, SD 7.88 β −0.019 SE 0.038 *p* 0.62
	No μ 12.29 SD 5.56	No median 92.93 IQR 14.9	No median <0.01 IQR 0.54	No median 0.01 IQR 0.01	No median <0.01 IQR 0.01	No median <0.01 IQR 0.01	No median <0.01 IQR <0.01	No μ 7.79 SD 6.19	No μ 12.96, SD 7.15
12. Grew up with dog	Yes μ 14.95 SD 11.46 –	Yes median 92.31 IQR 17.56 β 0.028 SE 0.039 *p* 0.47	Yes median 0.33 IQR 2.67 β −0.011 SE 0.03 *p* 0.72	Yes median 0.01 IQR 0.02 –	Yes median 0.01 IQR 0.01 –	Yes median <0.01 IQR 0.01 β 0.0055 SE 0.0044 *p* 0.21	Yes median <0.01 IQR <0.01 –	Yes μ 10.88 SD 11.61 β 0.0073 SE 0.025 *p* 0.77	Yes μ 9.94, SD 7.29 β −0.018 SE 0.022 *p* 0.41
	No μ 15.43 SD 10.44	No median 88.31 IQR 33.02	No median <0.01 IQR 1.45	No median 0.01 IQR 0.02	No median 0.01 IQR 0.01	No median <0.01 IQR 0.01	No median <0.01 IQR <0.01	No μ 8.27 SD 8.4	No μ 13.07, SD 9.41
13. Child/children at home	Yes μ 13.77 SD 12.09 –	Yes median 91.23 IQR 15.93 –	Yes median <0.01 IQR 2.35 –	Yes median 0.01 IQR 0.02 –	Yes median 0.01 IQR 0.01 –	Yes median <0.01 IQR 0.01 –	Yes median <0.01 IQR <0.01 –	Yes μ 11.33 SD 11.76 –	Yes μ 10.04, SD 6.58 –
	No μ 15.43 SD 10.98	No median 92.06 IQR 20.5	No median 0.33 IQR 2.52	No median 0.01 IQR 0.02	No median 0.01 IQR 0.01	No median <0.01 IQR 0.01	No median <0.01 IQR <0.01	No μ 10.05 SD 10.86	No μ 10.74, SD 8.2
14. Work with dogs	Yes μ 13.88 SD 11.68 –	Yes median 93.14 IQR 22.85 –	Yes median <0.01 IQR 1.45 β −0.048 SE 0.027 *p* 0.074	Yes median 0.01 IQR 0.02 –	Yes median <0.01 IQR 0.01 –	Yes median <0.01 IQR 0.01 –	Yes median <0.01 IQR <0.01 –	Yes μ 11.36 SD 12.1 –	Yes μ 9.59, SD 6.53 –
	No μ 15.49 SD 11.07	No median 91.42 IQR 17.77	No median 0.44 IQR 2.66	No median 0.01 IQR 0.02	No median 0.01 IQR 0.01	No median <0.01 IQR 0.01	No median <0.01 IQR <0.01	No μ 9.96 SD 10.66	No μ 10.95, SD 8.28

*Question 1, human gender, was reported in the previous study (41). Question 4 is a repetitive question of questions 2 and 9; also, high VIF values (VIF>2) were detected, so question 4 was excluded from the analysis.*

*Track (%): tracking time (s)/total walking time (s) × 100%. Tail high (%): tail high time (s)/total walking time (s) × 100%. Tail wag (%): tail wagging time (s)/total walking time (s) × 100%. Gaze (/sec): Numbers of gazes/time when the dog's head was visible in the Gopro video (s). Lip-lick (/sec): Numbers of lip-licks/time when the dog's head was visible in the Gopro video (s). Eliminate-mark (/sec): Numbers of eliminate-marks/total walking time (s). Shake (/sec): Numbers of shakes/total walking time (s). Pant (%): painting time (s)/time when the dog's head was visible in the Gopro video (s) × 100%. Sniff (%): sniffing time (s)/total walking time (s) × 100%.*

a *Analysed in power of 7.*

b *Analysed in power of 0.3.*

c *Analysed in power of 0.4.*

d *Analysed in power of 0.6.*

e *Analysed in power of 0.8.*

f *Analysed in power of 0.5.*

*Wagging tail, shaking body and sniffing were not entered into the generalised linear mixed model because both predictors, dog and human genders, had high p-values in the bivariate regression models. μ: mean (before transformation). SD, standard deviation of μ; IQR, interquartile range; β, regression coefficient; SE, standard error of β; p, p-value of the model. Question 5, educational level, secondary school was used for comparison. Question 6, relationship status, volunteers who were singled were used for comparison. Question 7, training level, level 3+ was used for comparison. Question 8, volunteering frequency, frequency greater than once a week was used for comparison. Only one participant volunteered fortnightly. To prevent bias, this participant was excluded from the analysis related to volunteering frequency. Question 9, volunteering time between 6 and 12 months was used for comparison. Only 1 volunteer had volunteering experience <1 month. To prevent bias, the person was excluded from analysis of volunteering time. Question 10, volunteers who was not living with a dog at the time of conducting the research were used for comparison. Question 11, volunteers who had not lived with a dog before the time of conducting the research were used for comparison. Question 12, volunteers who did not grow up with a dog were used for comparison. Question 13, volunteers who did not live with a child/children at the time of conducting the research were used for comparison. Question 14, volunteers who did not work with a dog at the time of conducting the research were used for comparison. –: Not included in the generalised linear mixed model because the predictor had high p-values in the bivariate regression model*.

### Human Demographics and Walking Experience

Volunteers' age was positively correlated with the score of factor H (*p* = 0.017), indicating a more positive reaction to the walk. Volunteers holding a postgraduate (*p* = 0.021) or bachelor's (*p* = 0.012) degree scored lower on factor H than those with secondary school as the highest educational level. Compared to level 3+ volunteers, level 2 (*p* = 0.0066) and 3 (*p* = 0.012) volunteers scored lower on factor H. Volunteers with 1–2 years of experience scored lower on factor H (*p* = 0.0075) compared to those with 6–12 months of volunteering experience (Table 9).

**Table 9:** Generalised linear mixed model of the effects of human demographics, human behaviour, maximal tension by dog (DT_max_), and mean tension by dog (DT_mean_) on volunteers' walking experience (factor H and factor D).

	**Factor H^**a**^**	**Factor D**
2. Age	β 55391 SE 23010 *p* 0.017	–
3. Height	–	–
5. Educational level	Postgraduate median 4.93 IQR 0.54 β −2268831 SE 976103 *p* 0.021	Postgraduate μ 4.38, SD 0.56 β 0.0016 SE 0.16 *p* 0.99
	Bachelor median 4.79 IQR 0.57 β −1752164 SE 689171 *p* 0.012	Bachelor μ 4.05, SD 0.67 β −0.2 SE 0.12 *p* 0.077
	Training college median 5.00 IQR 0.29 β −423689 SE 638822 *p* 0.51	Training college μ 4.32, SD 0.62 β −0.022 SE 0.1 *p* 0.83
	Secondary school median 5.00 IQR 0.43	Secondary school μ 4.38, SD 0.64
	Primary school median 4.86 IQR 0.39 β 351453 SE 1471890 *p* 0.81	Primary school μ 4.13, SD 0.71 β −0.43 SE 0.26 *p* 0.1
6. Relationship status	Single median 4.86 IQR 0.43	Single μ 4.3, SD 0.64
	Married/partnered median 5.00 IQR 0.43 β 529099 SE 567703 *p* 0.35	Married/partnered μ 4.31, SD 0.63 –
	Separated/divorced or widowed median 4.86 IQR 0.79 β −1319075 SE 1406575 *p* 0.35	Separated/divorced or widowed μ 4.33, SD 0.71 –
7. Training level	Level 2 median 4.86 IQR 0.57 β −1781341 SE 647316 *p* 0.0066	Level 2 μ 4.31, SD 0.67 –
	Level 3 median 4.86 IQR 1.00 β −1641509 SE 643957 *p* 0.012	Level 3 μ 4.18, SD 0.68 –
	Level 3+ median 5.00 IQR 0.29	Level 3+ μ 4.35, SD 0.61
8. Volunteering frequency	>Once a week median 5.00 IQR 0.43	> Once a week μ 4.4, SD 0.59
	Once a week median 4.86 IQR 0.57 –	Once a week μ 4.25, SD 0.67 β −0.066 SE 0.087 *p* 0.45
9. Volunteering time	> 2 years median 5.00 IQR 0.14 β −541583 SE 679079 *p* 0.43	> 2 years μ 4.36, SD 0.59 –
	1–2 year(s) median 4.86 IQR 0.79 β −1629054 SE 600660 *p* 0.0075	1–2 year(s) μ 4.26, SD 0.74 –
	6–12 months median 5.00 IQR 0.36	6–12 months μ 4.23, SD 0.63
	1–6 months median 4.86 IQR 0.5 β 420414 SE 581907 *p* 0.47	1–6 months μ 4.42, SD 0.58 –
10. Living with dog	Yes median 4.86 IQR 0.43 β −533037 SE 570993 *p* 0.35	Yes μ 4.34, SD 0.62 β −0.088 SE 0.092 *p* 0.34
	No median 4.86 IQR 0.75	No μ 4.23, SD 0.68
11. Lived with dog	Yes median 4.86 IQR 0.43 –	Yes μ 4.31, SD 0.64 –
	No median 4.86 IQR 0.57	No μ 4.31, SD 0.63
12. Grew up with dog	Yes median 4.86 IQR 0.43 β 810760 SE 599713 *p* 0.18	Yes μ 4.33, SD 0.65 β 0.082 SE 0.11 *p* 0.45
	No median 4.86 IQR 0.57	No μ 4.21, SD 0.6
13. Child/children at home	Yes median 4.86 IQR 0.57 –	Yes μ 4.27, SD 0.65 –
	No median 4.86 IQR 0.43	No μ 4.32, SD 0.64
14. Work with dogs	Yes median 5.00 IQR 0.29 β 406744 SE 534141 *p* 0.45	Yes μ 4.43, SD 0.57 β 0.013 SE 0.09 *p* 0.89
	No median 4.86 IQR 0.57	No μ 4.26, SD 0.66
Attention seeking (no./sec)	–	β −3.06 SE 1.89 *p* 0.11
Negative verbal cue (no./sec)	β −15842007 SE 39766630 *p* 0.69	β −14.75 SE 7.35 *p* 0.046
Praise (no./sec)	β 8891984 SE 8823625 *p* 0.32	β −0.26 SE 1.58 *p* 0.87
Command (no./sec)	–	β 2.065 SE 1.55 *p* 0.18
Hand gesture (no./sec)	–	β −4.97 SE 5.94 *p* 0.4
Physical contact (no./sec)	β 77293346 SE 36890086 *p* 0.038	β 6.65 SE 6.18 *p* 0.28
DLT _max_	β 86341 SE 177683 *p* 0.63	β −0.016 SE 0.031 *p* 0.6
DLT _mean_	β −1919702 SE 696659 *p* 0.0066	β −0.28 SE 0.11 *p* 0.011

*Human satisfaction factor (Factor H): A higher factor H score indicated that the handler was more satisfied with the interaction. Dog behaviour factor (Factor D): A higher factor D score indicated that the handler considered the dog better behaved.*

a *Analysed in power of 10.*

*Question 5, educational level, secondary school was used for comparison. Question 6, relationship status, volunteers who were singled were used for comparison. Question 7, training level, level 3+ was used for comparison. Question 8, volunteering frequency, frequency greater than once a week was used for comparison. Only one participant volunteered fortnightly. To prevent bias, this participant was excluded from the analysis related to volunteering frequency. Question 9, volunteering time between 6 and 12 months was used for comparison. Only 1 volunteer had volunteering experience <1 month. To prevent bias, the person was excluded from analysis of volunteering time. Question 10, volunteers who was not living with a dog at the time of conducting the research were used for comparison. Question 11, volunteers who had not lived with a dog before the time of conducting the research were used for comparison. Question 12, volunteers who did not grow up with a dog were used for comparison. Question 13, volunteers who did not live with a child/children at the time of conducting the research were used for comparison. Question 14, volunteers who did not work with a dog at the time of conducting the research were used for comparison. Total verbal cue, communication, high-pitched voice, total body language and food reward were not included in the model due to high p-values. μ: mean (before transformation). SD, standard deviation of μ; IQR, interquartile range; β, regression coefficient; SE, standard error of β; p, p value of the model. –: Not included in the generalised linear mixed model because the predictor had high p-values in the bivariate regression model*.

## Discussion

### Age

Cimarelli et al. have shown that older owners provide less social support to their dogs in stressful situations (15). However, in our study, older volunteers were found to talk to dogs (communicating, praising, and using commands) more often during the walk than younger volunteers. This may reflect the dog's orientation to baby-like talk which is more prominent in older volunteers (64, 65). Also, volunteers who were older enjoyed the interaction more, which supports a positive relationship between job satisfaction and workers' age (66). A possible explanation may be that older volunteers may have more experience of both volunteering and life in general, and thus may be better prepared to cope with challenges (67). They may also just be happy to enjoy life and accept opportunities to do so.

### Height

When walking with taller volunteers, dogs tended to pull on the leash more frequently. This was coupled with the negative relationships between height and communication time and the frequency of physical contacts, and more frequent use of high-pitched voices (getting attention) and commanding. The reduced physical contact may result from the greater physical distance between taller individuals and dogs. Also, taller individuals may be used to being seen as dominant and so may tend to command but not communicate (68). Dogs are more stressed and defensive when facing men than women possibly due to their greater height that is perceived as intimidating (25). However, our results did not find any correlation between canine stress-related signs (e.g., panting, lip-licking and tail in lower positions) and volunteers' height. Therefore, there might be characteristics other than height (e.g., humans' empathy, behaviour and dogs' previous experiences of interacting with humans) that cause shelter dogs displaying more stress related signals towards men.

### Educational Level

Volunteers with a postgraduate degree were more likely to praise dogs, give food treats, and tended to use more body language, including hand gestures and physical contact. Highly educated volunteers may have been more aware of different communication approaches, both verbal and non-verbal (69). Also, highly educated individuals are better at correctly identifying dogs' stress (17) and more likely to adopt reward based training (16). Perhaps they were just more prepared for walking dogs because they tend to read more and are more equipped with relevant knowledge.

People with higher educational levels have previously been reported as being close to their dogs (18). However, in our study, volunteers with a bachelor's or a postgraduate degree were less satisfied with the interaction and felt less supported, either emotionally or physically, by the dogs, which might result from the different nature of the human-dog relationship in our study. Differentiating it from previous studies investigating the relationship between owners and their own dogs (12, 26), our research focused on the interaction between shelter dogs and volunteers which is a relatively short-term relationship with weaker human-dog bonds. Another possible explanation may be that volunteers with lower educational levels were more likely to engage in higher levels of anthropomorphism which might facilitate their bonds with shelter dogs (40, 70).

### Relationship Status

Married and partnered volunteers tended to verbally praise dogs, while separated/divorced or widowed volunteers initiated more physical contacts with dogs. Like single mothers, separated/divorced or widowed volunteers may be more in need of emotional contact, which they seek to obtain from the dogs (similar to the role of children) through physical contact, compared with married/partnered individuals (71). Frequent physical contact then attenuates the stress levels of dogs, hence less panting in dogs walked by single, separated/divorced or widowed volunteers.

### Training Level

There was no correlation between leash tension/pulling frequency and volunteers' training level, except that net mean tension was lower in level 2 volunteers, probably because level 2 volunteers only walked well-behaved dogs (level 1 and 2 dogs). This confirms that leash tension created by dogs is largely determined by the dogs themselves (39). Similarly, no difference was observed between different volunteers' levels with respect to the tension and pulling frequency by handlers, probably because dogs were matched with volunteers. If dogs were paired with volunteers randomly, it would have been easier to detect any differences, but this was perceived to be unethical in our study.

Compared to level 3+ volunteers, level 3 (but not level 2) volunteers were more likely to talk to dogs, including communication, praise, using a high-pitched voice and commands, but less likely to initiate physical contact with dogs. Probably the level 3 volunteers had more opportunities than level 3+ volunteers to interact with well-behaved dogs, making them more likely to use positive verbal expressions. In addition, compared to level 2 volunteers, level 3 volunteers were more aware of reward-based interactions, while avoiding unnecessary physical contact that might intimidate or frighten the dogs. Level 3 volunteers might also be less experienced in handling different dogs, potentially making dogs more stressed, as evidenced by them wagging their tails less but panting more. This is supported by the fact that level 2 and 3 volunteers were generally less satisfied with the interaction than level 3+ volunteers and perceived the walks as more challenging. However, this should be interpreted with caution because volunteers from all levels did not score differently in terms of their feelings about the dogs' behaviours.

### Volunteering Frequency and Time

Compared to participants volunteering more than once a week, those volunteering only once every week verbally attracted the attention of dogs less often, probably because they had a weaker bond with the dogs. Compared with participants with 6–12 months of volunteering experience, those with 1–2 years of experience walked dogs that shook their bodies less, a sign of stress (72, 73). This is perhaps because those with longer volunteering experience had a better knowledge of correctly interacting with dogs. However, this finding should be interpreted with care as no difference was found in volunteers attending the shelter for more than 2 years, though it is possible that many of them did not make many improvements after gaining a certain level of experiences (74).

Compared to volunteers with 6–12 months of volunteering experience, those who had been volunteering at the RSPCA for 1–2 years were less satisfied with the interaction. There is little comparable evidence for volunteers in shelters, but nurses have a turnover rate of around 50% in the first year of employment due to the individual's inexperience and inability to deal with complicated situations (75). The turnover rate of animal shelter volunteers has not been clearly identified. However, from our data, the number of volunteers seemed to decline after 12 months of volunteering, since 31% of our volunteers had 6–12 months of volunteering experience and only 16.2 % had more than 2 years. Volunteers perhaps become less satisfied with their work and stop volunteering after 1–2 years. Euthanasia of animals (76), availability of professional development and the opportunity of developing role identity (74) have all been reported to affect volunteers' commitment. Future studies are warranted to investigate the turnover rate and working latency of volunteers working in animal shelters.

### Experiences of Living and Working With Dogs and Living With Children

If volunteers grew up with dogs, the dogs pulled less and the volunteers interacted with dogs using fewer words and less body language. This suggests a more benign relationship. Similarly, volunteers currently living with dogs were less likely to talk to shelter dogs using a high-pitched voice and the dogs pulled less. People may be less excited meeting new dogs if they have experience of dog ownership and may be more experienced in controlling dogs when they are on the leash (24, 73). However, volunteers who worked with dogs regularly were more talkative when walking shelter dogs. A possible explanation may be that these people were more dog oriented, being more extraverted, socially bold and talkative (77, 78). Finally, volunteers with a child or children at home were more likely to interact with dogs using body language, including hand gestures and physical contact, potentially because these volunteers were more sensitive to the needs of dogs, as if they were their children (73, 79). Also, they might be more aware of effective ways of communicating with dogs (similar to children with impaired verbal communication) and adapt their behaviours accordingly by using more physically directiveness (80).

### Application and Future Study

This study has identified some correlations between human demographics and their behavioural interactions with shelter dogs. Results may be used to improve the welfare of shelter dogs by matching them with suitable volunteers. For instance, dogs that tend to pull on the leash could be recommended to be walked by shorter volunteers who had/have owned dogs. Dogs that feel stressed and sensitive to physical contacts by humans may be walked by married/partnered volunteers who are taller and currently not living with kids, while dogs that enjoy interacting with humans may be suitable to partner with older volunteers. This is an exploratory study and by combining results of this and our previous papers (39, 41, 43, 44) and more future evidence identifying the characteristics of volunteers suitable for walking shelter dogs, shelters may be able to develop an improved human-dog combination that can benefit the animal welfare. Also, the results may help develop training program for all volunteers who wish to walk shelter dogs. For volunteers, this study has found that people who are younger, with a higher educational level and have volunteered for 1–2 years seem to be less satisfied with their interaction with dogs. Future study may investigate underlying reasons that influence people's experiences of volunteering in an animal shelter and shelters may modify their procedures to retain more volunteers.

### Limitations of the Study

One limitation of this study was that dogs were not randomly matched with participants. Dogs were assigned to participants based on their behaviours and participants' experience, due to safety and animal welfare concerns. In addition, the results were obtained from a single shelter in Queensland, Australia. Given the potential effects of people's cultural backgrounds on their interaction with dogs (81), more studies are needed in the future for broader generalisation.

## Conclusions

We found correlations between volunteers' demographics and the behavioural interactions. Human demographics included age, body height, educational level, marital status, training level, volunteering frequency and time and experiences of living and working with dogs and living with children. Our study might contribute to better working experience and animal welfare in a shelter by improving the matching of volunteers and shelter dogs. Pairing potential owners and dogs for more satisfying partnerships is another potential, though it was not the prime objective of this experiment and would need further research.

## Data Availability Statement

The raw data supporting the conclusions of this article will be made available by the authors, without undue reservation.

## Ethics Statement

The studies involving human participants were reviewed and approved by Human Research Ethics Committees (Approval numbers: 2018001570) of The University of Queensland. Written informed consent to participate in this study was provided by the participants' legal guardian/next of kin. The animal study was reviewed and approved by Animal Ethics Committees (Approval numbers: SVS/400/18, respectively) of The University of Queensland.

## Author Contributions

H-YS, CP, MP, and NP: conceptualisation, methodology, and writing—review and editing. H-YS: software, investigation, and writing—original draft preparation. H-YS and CP: validation, formal analysis, data curation, visualization, and funding acquisition. MP and CP: resources. CP, MP, and NP: supervision. H-YS, CP, and MP: project administration. All authors contributed to the article and approved the submitted version.

## Funding

This research was funded by the Higher Degree Research Fund of University of Queensland and the RSPCA Fund donated by philanthropists.

## Conflict of Interest

MP is employed as the principal scientist by RSPCA, QLD. The remaining authors declare that the research was conducted in the absence of any commercial or financial relationships that could be construed as a potential conflict of interest.

## Publisher's Note

All claims expressed in this article are solely those of the authors and do not necessarily represent those of their affiliated organizations, or those of the publisher, the editors and the reviewers. Any product that may be evaluated in this article, or claim that may be made by its manufacturer, is not guaranteed or endorsed by the publisher.
