# Supplementary material for: Volunteers' Demographics That Affect the Human-Dog Interaction During Walks in a Shelter
Source: Front Vet Sci. 2021 Sep 7;8:699332. doi: 10.3389/fvets.2021.699332 (PMC8452965; doi:10.3389/fvets.2021.699332)
Supplement: Supplementary file 1 [file Table_1.DOCX]

Supplementary Material

**Appendix table 1. Demographic questionnaire for volunteers (n = 74) following walking dogs (n = 111) on a designated route at RSPCA Queensland.**

| 1. What is your gender?  [ ] Male [ ] Female [ ] Other |
| --- |
| 2. What is your current age?  [ ] |
| 3. What is your height?  [ ] cm |
| 4. What was your age when you began to volunteer with dogs regularly?  [ ] |
| 5. What is your highest educational level?  [ ] Primary school [ ] Secondary school [ ] Training college (TAFE) [ ] Bachelor’s  degree [ ] Postgraduate degree |
| 6. What is your relationship status?  [ ] Married/partnered [ ] Single [ ] Separated/divorced or Widowed |
| 7. What is the highest level of RSPCA dog-walking training you have completed?  [ ] 1 [ ] 2 [ ] 3 [ ] 3+ |
| 8. How often do you volunteer?  [ ] More than once a week [ ] Once a week [ ] Once a fortnight [ ] Less than once  a fortnight |
| 9. How long have you been volunteering in RSPCA?  [ ] < 1 month [ ] 1-6 months [ ] 6-12 months [ ] 1-2 year(s) [ ] > 2 years |
| 10. Are you currently living in a household with a dog?  [ ] Yes [ ] No |
| 11. Have you previously lived in a household with a dog?  [ ] Yes [ ] No |
| 12. Did you grow up with a dog in your household?  [ ] Yes [ ] No |
| 13. Do you have a child/ children living in your home?  [ ] Yes [ ] No |
| 14. Do you work in an area that deals with dogs on a regular basis?  [ ] Yes [ ] No |
